# Evaluating pituitary tumor management: aligning with pituitary tumor centers of excellence criteria

**DOI:** 10.1007/s11102-025-01563-8

**Published:** 2025-08-14

**Authors:** Jin-Han Yang, Wei-Hsin Wang, Harn-Shen Chen

**Affiliations:** 1 Division of Endocrinology and Metabolism, Department of Medicine, Taipei Veterans General Hospital, Taipei, Taiwan; 2 Department of Neurosurgery, Taipei Veterans General Hospital, Taipei, Taiwan; 3 School of Medicine, National Yang Ming Chiao Tung University, Taipei, Taiwan

## Abstract

**Background:**

The Pituitary Society established criteria to develop Pituitary Tumor Centers of Excellence (PTCOE) for optimization of patient care. These criteria were later transformed into quantitative standards based on real-life data from accredited pituitary centers worldwide. The aim of this study was to evaluate the pituitary tumor care capacity at our institute and compare it with the PTCOE criteria.

**Method:**

We retrospectively reviewed the data of patients who underwent sellar, suprasellar, or parasellar surgery during 2021–2023 at Taipei Veterans General Hospital. Adults older than 18 years who were diagnosed with pituitary tumors were included. We collected data regarding baseline patient and tumor characteristics, the surgical method, complications, and activity volumes across involved departments. The relevant data were compared with those of the PTCOE standards.

**Results:**

In total, 182 patients with pituitary tumors underwent surgery via the endoscopic endonasal approach during 2021–2023. Their median age was 51 (range, 20–91) years. Among them, 90.7% had macroadenomas. Functional remission rates were 55.6% for acromegaly, 69.2% for prolactinoma, and 93.3% for Cushing’s disease. Our institute met the acceptable PTCOE criteria for the number of pituitary interventions, postoperative readmissions for complications, dynamic endocrine tests, neuroradiologists, and neuro-oncologists. Further, we met the preferred PTCOE criteria for mortality rate and the numbers of dedicated surgeons, endocrinologists, trained nurses, neuropathologists, and neuro-ophthalmologists.

**Conclusion:**

Most indicators in our study met the acceptable standards for a PTCOE. During the study period, the multidisciplinary team at our institute collaborated closely to provide comprehensive care for patients with pituitary adenomas.

## Introduction

Pituitary adenomas are usually benign; however, affected patients may have comorbidities that influence their quality of life and further reduced their life expectancy [1]. Therefore, diagnosis and appropriate individualized management are important.

Owing to the low incidence of pituitary adenomas and the need for complex interventions, the concept of “centers of excellence” has been proposed to ensure more specialized management and optimal patient care for this condition. In 2017, the Pituitary Society generated criteria for the development of a PTCOE, in which the general characteristics and mission of PTCOEs were defined [2]. Later, Giustina et al. investigated nine pituitary centers with a worldwide reputation to survey and evaluate the activity in the pituitary field. They transformed the definition of an accredited PTCOE from qualitative parameters to quantitative criteria according to real-world data [3].

With the increase in awareness of care for patients with pituitary tumors, a comprehensive investigation of such care at our institute became necessary. In this study, we aimed to investigate our pituitary tumor care capacity by collecting the numerical data associated with pituitary adenoma surgery and the numbers of involved specialists. We also aimed to compare our real-life circumstances with the published criteria for PTCOEs.

## Method

We retrospectively reviewed the medical charts of patients who underwent sellar, suprasellar, or parasellar surgery from Jan 1 st, 2021, to Dec 31 st, 2023, at Taipei Veterans General Hospital. Patients who were older than 18 years and were diagnosed with pituitary tumors were included. Patients with diagnoses other than pituitary tumors were excluded. Patient baseline characteristics, tumor characteristics, tumor-related symptoms, functional examinations, operative method, surgical complications, and post-operative hormone insufficiency were recorded. We also gathered information on personnel and activity volume from other supporting units at our hospital. This study was based in part on data from the Big Data Center, Taipei Veterans General Hospital. The interpretation and conclusions contained herein do not represent the position of the hospital. The study was approved by the institutional review board of the hospital under approval code 2025-02-001AC, and performed in compliance with the tenets of the Declaration of Helsinki.

Postoperative biochemical remission or functional remission was defined as follows: for acromegaly, normalization of IGF-1 concentration according to the patient’s age at 12 weeks after the surgery [4]; for Cushing’s disease, postoperative serum cortisol concentrations lower than 2 µg/dL when monitored until postoperative cortisol nadir [5]; for prolactinoma, postoperative normalization of prolactin serum levels [6]; and for gonadotroph adenomas or other non-functioning pituitary adenomas (NFPAs), recovery from disease-induced abnormal hormone secretion 4–8 weeks after surgery, without the need for hormone replacement in patients who had had pituitary hormone deficiencies before surgery [7, 8]. Postoperative hypopituitarism was diagnosed according to laboratory tests or symptoms related to hormonal deficiency, and the use of hormone replacement therapy. Major complications were defined as mortality related to complications of the surgery, the need for re-operation, or impairments in physical function within 30 days postoperatively. Long-term hypopituitarism was defined as the need for hormone replacement therapy for more than 3 months after the operation.

Descriptive statistics were used to present case counts. Continuous variables were presented as means and standard deviations. Statistical analysis was performed using IBM SPSS Version 25.0 (IBM Corp., Armonk, NY, USA).

## Results

**Fig. 1:**
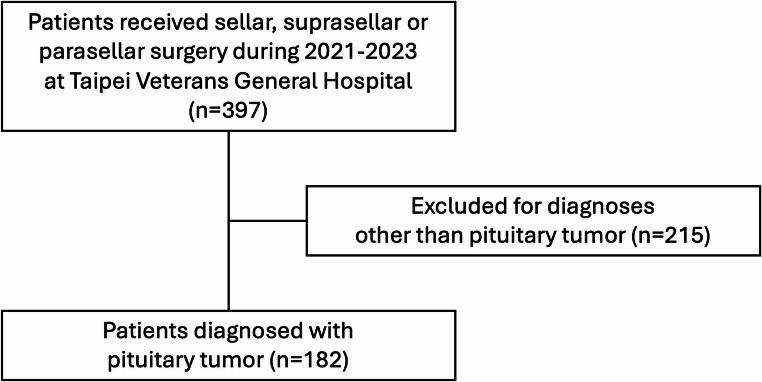
Flow chart of patient selection process

In total, 397 patients underwent sellar, suprasellar, or parasellar surgery during 2021–2023 at Taipei Veterans General Hospital. Of these, 215 patients were excluded owing to diagnoses other than pituitary tumors (Fig. 1). The remaining 182 patients underwent endoscopic endonasal approach (EEA) for pituitary tumor removal during the study period, males accounting for 54.4% of the sample. Their median age was 51 (range: 20–91) years. Five patients underwent two surgical interventions, two of which because of postoperative complications, two because of experiencing tumor recurrence, and one because of a residual tumor. Therefore, a total of 187 EEA surgical interventions were performed during the reviewing period. Macroadenomas accounted for 90.7% of tumors, and the most common tumor type was gonadotroph adenoma. In total, 105 patients experienced optic chiasm compression before the operation, of whom 86 (81.9%) had postoperative vision improvement. Eighteen patients underwent postoperative Gamma Knife surgery.

Functional remission after the operation was achieved in 55.6% of patients with acromegaly, 93.3% with Cushing’s disease, and 69.2% with prolactinoma. 40% of the patients with NFPAs still required hormone replacement after the operation.

The core activity data and the personnel involved in the treatment of patients with pituitary tumors at our hospital are listed in Table 1. In total, 62 pituitary interventions were done per year in average. The mortality rate was 0.2%, which was not related to the operation but was due to refractory somatotroph tumor disease. 770 dynamic endocrine tests were performed per year during the study period. Dexamethasone suppression tests were performed most frequently, with an average of 453 tests per year. Inferior petrosal sinus sampling was performed five times per year in average. The average annual number of pituitary CT scans was 55, and that of pituitary contrast MRI scans was 1162.

**Table 1:** Core activity data and activity volumes at our Institute

	Number per year
Neurosurgery unit	
Pituitary interventions	62
Patients with complications requiring inpatient readmission within 30 days (%)	7 (3.8%) ^1^
Mortality (%)	0.2% ^2^
Personnel	
Dedicated surgeons	5
Trained nurses	12
Trained technicians	16
Endocrinology unit	
Dynamic examinations	770
Personnel	
Endocrinologists	7
Trained nurses/technicians	12
Supporting units	
Neuroradiologists	2
Sellar MRIs and CT scans	1217
Sellar MRIs	1162
IPSS cases	5.3
Radiation therapy (gamma knife) cases	6
Neuropathologists	3
Neuro-oncologists	2
Neuro-ophthalmologists	2

^1^ Complications either requiring re-operation or resulting in impairment of physical function

^2^ The one who had refractory disease of somatotroph tumor despite re-operation for recurrence tumor removal and medication treatment

PTCOE, Pituitary Tumor Center of Excellence

IPSS, inferior petrosal sinus sampling

## Discussion

In this study, we comprehensively examined the core activities related to pituitary tumors at our hospital. We found that most items were within the acceptable range for PTCOEs according to published recommendations. The mortality rate, numbers of dedicated surgeons, endocrinologists, trained nurses, neuropathologists, and neuro-ophthalmologists also met the preferred criteria.

The introducing of multidisciplinary team in management of pituitary adenoma has been shown to have benefits. Previous studies have shown shorter hospital stay, less intrasellar residual tumor, less complication rate, and decreased readmission rate after implementing of pituitary multidisciplinary team [9–12]. A proportion of these patients require medical therapy or radiotherapy after the operation. For instance, patients with growth-hormone secreting adenoma had post-operative biochemical remission rate of 51%−73.1% [13–15]. Patients with acromegaly or Cushing’s disease who cannot achieve biochemical control through medical treatment alone may require additional therapeutic interventions [16, 17]. Therefore, it is crucial that the endocrinology unit and other supporting units conduct long-term follow-up and provide ongoing management for such patients. Our institute had the preferred number of endocrinologists and trained nurses or technicians for a PTCOE during the study period. An acceptable number of dynamic endocrine tests were performed annually. Notably, more dexamethasone suppression tests were conducted than ACTH stimulation tests, likely owing to their convenience in an outpatient setting. Additionally, the capacity of the other supporting units at our institute met the acceptable criteria for a PTCOE.

The major surgical-complication rate in our study was 3.8%, meeting the acceptable criteria (< 10%) for a PTCOE [3]. This result corresponds with a multicenter analysis of 1240 patients who underwent transsphenoidal surgery, 6.9% experienced major complications, and the mortality rate was 0.7% [18]. Hypopituitarism developed in 11.0% of patients, most cases involving anterior hypopituitarism that required both cortisol and thyroxine replacement therapy. In previous studies, anterior pituitary insufficiency rates of 19% and diabetes insipidus rates of 18% were reported, with hypopituitarism rates varying from 5 to 31% depending on the neurosurgeon’s experience and tumor characteristics [19–22].

In this report, we examined the capacity of pituitary tumor care at our hospital. The multidisciplinary team at our center collaborated closely to provide comprehensive care for patients with pituitary tumors.

## Data Availability

No datasets were generated or analysed during the current study.
